# Gertrude Stein: A Physician Who Wasn’t to Be

**DOI:** 10.14797/mdcvj.1149

**Published:** 2022-09-06

**Authors:** James B. Young

**Affiliations:** 1 Academic Affairs, Cleveland Clinic, US; 2 Cleveland Clinic Lerner College of Medicine of Case Western Reserve University, Cleveland, Ohio, US; 3 Poet’s Pen, Methodist DeBakey Cardiovascular Journal, US

## Abstract

After a tumultuous time in the United States, including flunking out of medical school in 1901, Gertrude Stein, an iconic American author, art lover, and critic, moved to Paris in 1903 as an avant garde modernist who became a leading and legendary guru in the Parisian art and literature world.

## Stanzas in Meditation

### Stanza 1

I caught a bird which made a ball
And they thought better of it.
But it is all of which they taught
That they were in a hurry yet
In a kind of a way they meant it best
That they should change in and on account
But they must not stare when they manage
Whatever they are occasionally liable to do
It is often easy to pursue them once in a while
And in a way there is no repose
They like it as well as they ever did
But it is very often just by the time
That they are able to separate
In which case in effect they could
Not only be very often present perfectly
In each way whichever they chose.
All of this never matters in authority
But this which they need as they are alike
Or in an especial case they will fulfill
Not only what they have at their instigation
Made for it as a decision in its entirety
Made that they minded as well as blinded
Lengthened for them welcome in repose
But which they open as a chance
But made it be perfectly their allowance
All which they antagonize as once for all
Kindly have it joined as they mind

Gertrude Stein
Stanza I from *Stanzas in Meditation and Other Poems* (Los Angeles: Sun and Moon Press, 1994)

Permission to reprint was granted by the Estate of Gertrude Stein, through its Literary Executor, Mr. Stanford Gann, Jr. of Levin Gann PA.

## Gertrude Stein: A Physician Who Wasn’t to Be

As the Gilded Age came to an end and the Roaring Twenties emerged, the arts in general continued to evolve. Literature saw iconoclasts shaking up the written word and Cubist paintings bemused viewers. After a tumultuous time in the United States, including flunking out of medical school in 1901, Gertrude Stein, an iconic American author, art lover, and critic, moved to Paris in 1903 to be with her brothers, Michael and Leo.^1^ She made a few trips back to America but for the most part remained in Neully-sur-Seine, France, until her death in 1946, just after World War II.

**Figure:**
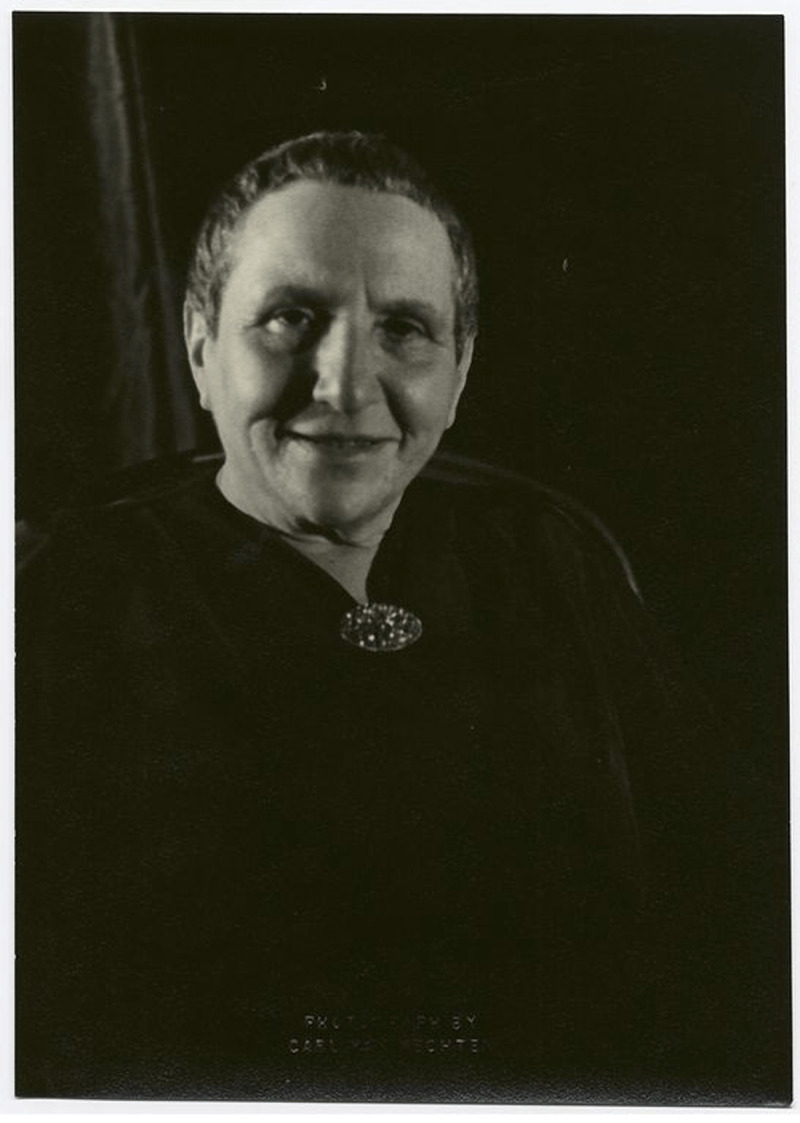
Gertrude Stein, New York, November 4, 1934. Henry W. and Albert A. Berg Collection of English and American Literature, The New York Public Library.

Stein was an avant-garde modernist who became a leading and legendary guru in the Parisian art and literature world. Her Paris salon at 27 rue de Fleurus, which she shared with her life partner and secretary, Alice B. Toklas (of “brownie” fame), became a destination for the “lost generation”—those intellectuals, artists, and authors who had fought in the world wars and subsequently were captivated by the “Movable Feast” and bohemian culture. These wanderers included artists Matisse, Picasso, Gris, Braque, Cezanne (Gertrude and her brother were some of the first collectors of the Cubist school), and authors such as Santayana, Hemingway, Fitzgerald, and Joyce (with their famous feuds).

Though committing to an artistic life as an author, arguably her only truly successful work was *The Autobiography of Alice B. Toklas*, a remembrance of Stein’s life written in more traditional prose using the “Toklas autobiography” as an artistic trick. Her poetry and other experimental writings were difficult to understand, yet they influenced subsequent generations of writers. Edmund Wilson, an American literary critic of the time, commented that, “Most of us balk at her [Stein’s] soporific rigmaroles, her echolaliac incantations, her half-witted-sounding catalogues of numbers….” Nonetheless, she remains often quoted with statements such as “A rose is a rose is a rose,” “What is the answer [In which I was silent] In that case what is the question?” and “There is no there there,” referring to California.^1^ Indeed, Stein became a beacon for artistic and bohemian communities worldwide. How did this happen, and what was her relationship to William Osler?

Gertrude Stein was born in Allegheny, Pennsylvania, in 1874 to a peripatetic family. She spent her early years living in Vienna, Paris, and eventually Oakland, California, where she came of age. Her father, involved with a San Francisco cable car company, seemed reasonably well off. She matriculated to the “Harvard Annex,” which became Radcliffe College while she was there (Harvard did not accept women at the time).^2,3,4^ Gertrude studied philosophy under George Santayana and psychology with William James, sometimes called the “father of psychology.”

Stein’s interests focused on the unconscious mind and automatic writing, or psychographics, which is the creation of texts and images without consciousness and perhaps being spiritually influenced. This technique appears to have directed much of her subsequent literary work, making it inscrutable at times. Though her work was published, she was influenced by James to attend medical school.^5,6^ Misogyny was rampant at the time, making it difficult for women to participate in that course of study.^7^ However, by philanthropic codicil, the recently opened Johns Hopkins School of Medicine in Baltimore was required to admit women as part of the student body. Stein entered with the class of 1897 and matriculated through several years of the curriculum. A class picture in 1901 shows 43 men and seven women, with Stein appearing detached and withdrawn in the back row. Five women dropped out during the course of study over the years. Stein made it to the last year, but apparently became distracted by her personal life and disinterested in the profession along the way. After failing several courses and refusing to take her final exams, she was either dismissed from the curriculum by William Osler or simply chose to leave Johns Hopkins School of Medicine on her own.

Several speculations have been published about events leading to her departure.^2,3,4^ Some attempted to place them in the context of the early 1900s related to women in medicine and, more generally, in society. Osler was accused of misogyny, for example, when judged by contemporary standards. Ultimately, the result was that Gertrude Stein did not become a medical doctor. But her medical training and research, particularly in the realm of psychology, seem to have influenced her poetry and other wordsmithing.

Gertrude Stein’s Stanza 1 in *Stanzas in Meditation* is an example of the work in its entirety and one of her most difficult poems to read and understand. It is complicated, disjointed, and abstract. It can be fun to read while trying to figure out the work’s meaning, but it can be frustrating as well, serving as an invitation to dig deeper into Stein’s other works and adventures. *Stanzas* emerged in the early 1930s, around the same time as her masterpiece, *Autobiography*, which was written in 1932 and quite different. It should be enjoyed by parsing out the words, short quips, and individual lines. Also, one might speculate on how much of the poem is actualized “automatic writing” by an individual who almost became a physician.
